# Sophocarpine alleviates doxorubicin-induced heart injury by suppressing oxidative stress and apoptosis

**DOI:** 10.1038/s41598-023-51083-3

**Published:** 2024-01-03

**Authors:** Hong-jin Zhang, Yang Fu, Huang Zhang, Ze-qun Lai, Yi-Fei Dong

**Affiliations:** 1 Department of Cardiovascular Medicine, The Second Affiliated Hospital of Nanchang University, China. No. 1 Minde Road, Nanchang, 330006 Jiangxi China; 2 Key Laboratory of Molecular Biology in Jiangxi Province, China. No. 1 Minde Road, Nanchang, 330006 Jiangxi China

**Keywords:** Pharmacology, Cardiovascular biology, Cardiovascular diseases

## Abstract

Doxorubicin (DOX) is an effective anti-tumor drug accompanied with many side effects, especially heart injury. To explore what effects of sophocarpine (SOP) on DOX-induced heart injury, this study conducted in vivo experiment and in vitro experiment, and the C57BL/6J mice and the H9C2 cells were used. The experimental methods used included echocardiography, enzyme-linked immunosorbent assay (ELISA), dihydroethidium (DHE) staining, terminal deoxynucleotidyl transferase dUTP nick end labeling (TUNEL) staining, western blotting and so on. Echocardiography showed that SOP alleviated DOX-induced cardiac dysfunction, as evidenced by the improvements of left ventricle ejection fraction and left ventricle fractional shortening. DOX caused upregulations of creatine kinase (CK), creatine kinase-MB (CK-MB) and lactate dehydrogenase (LDH), while SOP reduced these indices. The relevant stainings showed that SOP reversed the increases of total superoxide level induced by DOX. DOX also contribute to a higher level of MDA and lower levels of SOD and GSH, but these changes were suppressed by SOP. DOX increased the pro-oxidative protein level of NOX-4 while decreased the anti-oxidative protein level of SOD-2, but SOP reversed these effects. In addition, this study further discovered that SOP inhibited the decreases of Nrf2 and HO-1 levels induced by DOX. The TUNEL staining revealed that SOP reduced the high degree of apoptosis induced by DOX. Besides, pro-apoptosis proteins like Bax, cleaved-caspase-3 and cytochrome-c upregulated while anti-apoptosis protein like Bcl-2 downregulated when challenged by DOX, but them were suppressed by SOP. These findings suggested that SOP could alleviate DOX-induced heart injury by suppressing oxidative stress and apoptosis, with molecular mechanism activating of the Nrf2/HO-1 signaling pathway.

## Introduction

Doxorubicin (DOX) is an effective anti-tumor drug with strong therapeutic effects in the treatment of multiple cancers, so it was widely used for the treatment of multiple cancers^1^. However, it’s clinical application is limited by dose-dependent side effects, especially like heart injury, which may result in irreversible injury of heart and ultimately lead to heart failure^2^. Epidemiological information showed that the patients who accumulated DOX greater than 500 mg/m^2^, the incidence of heart failure is about 26%. When DOX reached 700 mg/m^2^, the incidence of heart failure increases into 48%^3^. Thus, searching an effective treatment for DOX-induced heart injury is of great significance.

The pathological changes of DOX-induced heart injury are complex, such as oxidative stress, inflammatory response, endoplasmic reticulum stress, DNA damage, apoptosis, autophagy, mitochondrial dysfunction, disturbed calcium homeostasis, and fibrosis^4–9^. Among them, oxidative stress and apoptosis have been demonstrated to be important factors for the development of DOX-induced heart injury^10,11^. Therefore, alleviating oxidative stress and apoptosis may be a promising strategy for the treatment of DOX-induced heart injury.

Sophocarpine (SOP), a quinolizidine tetracyclic alkaloid, which was extracted from sophora flavescens, has been suggested to have anti-nociceptive, anti-inflammatory, anti-oxidative stress, neuroprotective, immune regulatory functions, and anti-tumor activities^12^. Our previous study have showed that SOP has a protective effect on sepsis-induced heart injury^13^. However, it is unclear whether SOP exerts a cardioprotective effect against DOX-induced heart injury. The objective of this study was to investigate the effect of SOP on DOX-induced heart injury.

## Materials and methods

### Ethical approval

This investigation conforms to the *Guide for the Care and Use of Laboratory Animals* published by the US National Institutes of Health (NIH Publication No. 85–23, revised 1985). All the in vivo investigations were authenticated by the Animal Care and Use Committee of the Second Affiliated Hospital of Nanchang University (China) (application approval number = NCULAE-20221031143). This study was carried out in compliance with the ARRIVE guidelines.

### Mice and reagents

Twenty-four male C57BL/6J mice, which were purchased from Hunan SJA Laboratory Animal Co., Ltd., were applied to the in vivo experiment. Each mouse weighs about 25 g and is about 7 weeks old. Besides, the present study further conducted the in vitro experiment and the H9C2 cells were used in the in vitro experiment. The H9C2 cells were obtained from Chinese Academy of Medical Science. The mian reagents used in the present study included sophocarpine and doxorubicin. Sophocarpine (6483-15-4) was purchased from MedChemExpress Co., Ltd., with the purity be equal or greater than 98%. Doxorubicin (25316-40-9, DOX, Sigma Co., Ltd., USA) was applied to constructing heart injury model in vivo experiment and in vitro experiment.

### Mice model

**Figure 1:**
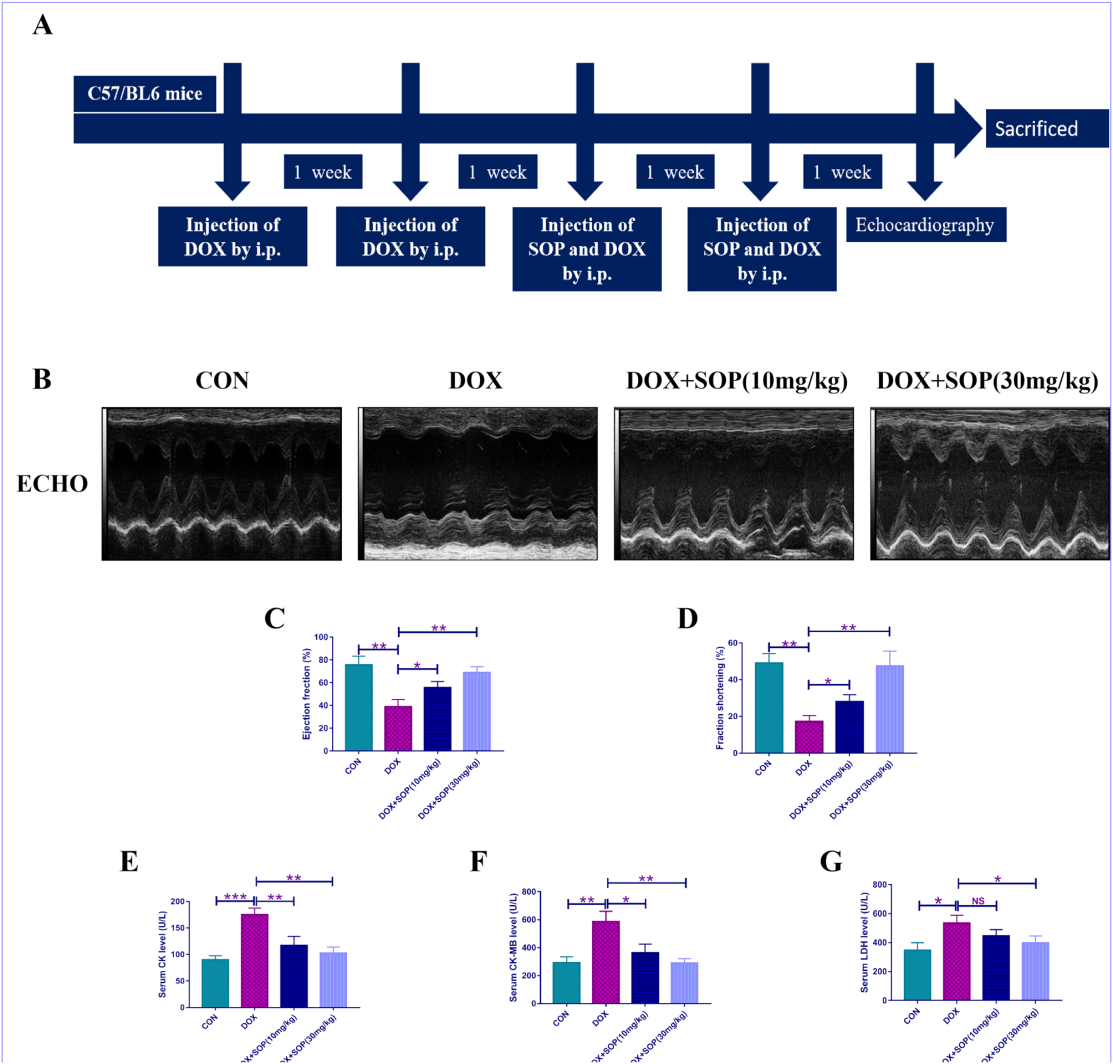
(**A**) An protocol of animal treatments in this study (n = 6). (**B**) The echocardiography was performed to evaluate cardiac function of the mice (n = 6). (**C**) and (**D**) the ejection fraction (%) and the fractional shortening (%) of left ventricle were acquired by automatic calculations mode (n = 6). (**E**–**G**) the serum levels of heart injury biomarkers (creatine kinase (CK), lactate dehydrogenase (LDH) and creatine kinase-MB (CK-MB)) were detected (n = 6). NS means no statistically significant; *p < 0.05, **p < 0.01 and ***p < 0.001. Data represent the mean ± SD of at least 3 times of separate experiments.

The mice used in the present study were fed in the Animal Center of Jiangxi Medical College of Nanchang University, with free access to the food and the water and adapting to the housing environment for about 1 week. The temperature and the humidity of the feeding environment stayed at 23 ± 2 °C and 53 ± 2%. These mice applied to the present study were randomly divided into four groups: CON group (n = 6): the mice in the CON group were treated with normal saline intraperitoneally (i.p.) once a week for 4 weeks and no other special treatment. Dox group (n = 6): the mice in the DOX group were treated with DOX (5 mg/kg) intraperitoneally once a week for 4 weeks and no other special treatment. DOX + SOP (10 mg/kg) (n = 6): first, the mice in the DOX + SOP (10 mg/kg) group were treated with DOX (5 mg/kg) intraperitoneally once a week for the first 2 weeks; then, DOX (5 mg/kg) accompany with SOP (10 mg/kg) were injected intraperitoneally into the mice together once a week for the last 2 weeks. DOX + SOP (30 mg/kg) (n = 6): first, the mice in the DOX + SOP (30 mg/kg) group were treated with DOX (5 mg/kg) intraperitoneally once a week for the first 2 weeks; then, DOX (5 mg/kg) accompany with SOP (30 mg/kg) were injected intraperitoneally into the mice together once a week for the last 2 weeks. The protocol of mice model was showed in Fig. 1A.

### Echocardiography

At the begining, the present study applied 2% isoflurane to anesthetizing these mice and fixed these mice on a heating board with supine-face up. Second, the device named Vevo770, which was purchased from VisualSonics Co., Ltd. of Canada, was used to perform transthoracic echocardiography and further assessed the cardiac function of these mice. In addition, the M-mode tracings short-axis view of this device was applied to measuring internal dimensions of left ventricle at the end-diastole stage and the end-systole stage. Finally, the left ventricle ejection fraction (LVEF %) and the left ventricle fractional shortening (LVFS %) were obtained automatically from this device.

### Heart injury biomarkers detection

Creatine kinase (CK), lactate dehydrogenase (LDH) and creatine kinase-MB (CK-MB) were common heart injury biomarkers. These mice were sacrificed by anaesthetization, with 3% pentobarbital sodium injection into the mice. The bloods were collcted to extract the serums, with a centrifugation at 4000 rpm for about 15 min. A device named Chemray240 automatic biochemical analyzer, which was obtained from Rayto Life Technology Co., Ltd. of China, was applied for measuring the serum levels of heart injury biomarkers. Besides, the heart tissues were collected in − 80 °C refrigerator for further investigations.

### Dihydroethidium (DHE) staining

The present study used DHE staining to assess the degrees of reactive-oxygen-species (ROS) in the heart tissues of the mice. Firstly, the paraffin slices of heart tissues were made; Secondly, these paraffin slices of heart tissues were incubated with DHE solution at 37 ℃ for about 40 min. Besides, the DAPI solution (1 mg/mL) was applied to counterstaining cell nucleus. Finally, these paraffin slices were washed by water and observed by a laser-scanning confocal microscope.

### Measurement of serum levels of oxidative stress biomarkers

The serum levels of oxidative stress biomarkers, like malondialdehyde (MDA) and superoxide dismutase (SOD) and glutathione (GSH) were detected by relevant kits. The informations of these relevant kits were as follows: MDA (A003-1, Nanjing Jiancheng Bioengineering Institute, China), SOD (A001-3, Nanjing Jiancheng Bioengineering Institute, China) and GSH (A006-2-1, Nanjing Jiancheng Bioengineering Institute, China). The procedures of assays were in line with the instructions provided by the manufacturers.

### Terminal deoxynucleotidyl transferase dUTP nick end labeling (TUNEL) staining

The paraffin slices of heart tissues of the mice were made. Next, the present study applied a specific fluorescent determination kit, which was purchased from Roche Co., Ltd. of China, for conducting TUNEL staining. The detailed informations of TUNEL staining were in accrdance with the instructions provided by the manufacturer.

### H9C2 cells treatments

The H9C2 cells were cultured with Dulbecco’s Modified Eagle’s Medium (DMEM), which contain 10% fetal bovine serum and 1% penicillin/streptomycin. The culture environment was maintained at appropriate parameters, with the concentrations of CO_2_ at 5% and the temperature at 37 ℃. Then, the H9C2 cells were pretreated by sophocarpine (1 μM, 2 μM, 5 μM) for 24 h and then challenged by DOX (1 μg/ml) for 12 h. Next, these H9C2 cells were randomly divided into 5 groups: CON group: the H9C2 cells in the CON group were treated with normal saline and no other special treatment. DOX group: the H9C2 cells in the DOX group were treated with DOX (1 μg/ml) for 12 h and no other special treatment. DOX + SOP (1 μM): the H9C2 cells in the DOX + SOP (1 μM) group were pretreated by sophocarpine (1 μM) for 24 h and then challenged by DOX (1 μg/ml) for 12 h. DOX + SOP (2 μM): the H9C2 cells in the DOX + SOP (2 μM) group were pretreated by sophocarpine (2 μM) for 24 h and then challenged by DOX (1 μg/ml) for 12 h. DOX + SOP (5 μM): the H9C2 cells in the DOX + SOP (5 μM) group were pretreated by sophocarpine (5 μM) for 24 h and then challenged by DOX (1 μg/ml) for 12 h.

### Detection of the degree of reactive-oxygen-species (ROS) in H9C2 cells

The present study used the ROS assay kit, which was purchased from Beyotime Biotechnology Co., Ltd. of China, to evaluate the degree of ROS in H9C2 cells. The density of the H9C2 cells was stayed at 1 × 10^5^ cells per well. Besides, the H9C2 cells were cultured in an appropriate environment for 24 h, with the concentrations of CO_2_ at 5% and the temperature at 37 ℃. Next, the old medium was replaced with the fresh medium, which added with 10 μM of DCFH-DA. Then, the H9C2 cells were cultured for 15 min. Finally, the H9C2 cells were washed for 3 times and the fluorescence microscope was applied for observing the H9C2 cells fluorescence intensity.

### Western blotting

The total protein was extracted from heart tissues of the mice by protein extraction kit, which was purchased from Beyotime Biotechnology Co., Ltd. of China. Then, the total protein was divided into different target proteins by 10% sodium-dodecyl sulfate–polyacrylamide gel-electrophoresis (SDS-PAGE). Next, these target proteins were transferred into the PVDF-membranes, which were obtained from EMD-Millipore Co., Ltd. of USA. The certain PVDF-membranes were incubated with primary antibodies for more than 8 h at 4 ℃, then incubated with secondary antibodies for 1 h at room temperature. Finally, a scanner and an advanced chemiluminescence solution were used to detect these target protein bands. It should be noted that the blots in the present study were cut prior to hybridisation with antibodies during the western blotting. The informations of these primary antibodies were as follows: anti-NOX-4 (ab154244, abcam, USA), anti-SOD-2 (CST-13141S, Cell Signaling Technology, USA), anti-Nrf2 (ab92946, abcam, USA), anti-HO-1 (ab52947, abcam, USA), anti-Bax (ab32503, abcam, USA), anti-Bcl-2 (ab182858, abcam, USA), anti-cleaved-caspase 3 (CST-9661S, Cell Signaling Technology, USA), anti-Cyto-C (ab133504, abcam, USA), anti-GAPDH (60004-1-Ig, Proteintech Rosemont, USA). The informations of these secondary antibodies were as follows: goat anti-mouse-IgG (15014, Proteintech Rosemont, USA) and goat anti-rabbit-IgG (B900210, Proteintech Rosemont, USA). The Image-Lab (version:4.0.1) software was used to analyze these data of target protein bands.

### Statistical analyses

All the results obtained from the present stuudy were showed as means and standard deviations (means ± SD). Besides, GraphPad Prism (v.7.0) software was applied to analyzing all of these results in the present study. In addition, the present study used analysis of variance (ANOVA) method to compare between different group. *p < 0.05, **p < 0.01 and ***p < 0.001 revealed that the difference was statistical significant.

## Results

### SOP alleviated DOX-induced heart injury in mice

The cardiac function was evaluated through echocardiography. Compared with the CON group, DOX markedly lead to heart injury in the DOX group, while SOP significantly improved DOX-induced heart injury in the DOX + SOP (10 mg/kg) group and the DOX + SOP (30 mg/kg) group, demonstrated by the improvements in LVEF (%) and LVFS (%) (Fig. 1B–D). In addition, the serum levels of heart injury biomarkers, such as creatine kinase (CK), creatine kinase-MB (CK-MB) and lactate dehydrogenase (LDH), upregulated apparently in the DOX group compared to the CON group, while them downregulated significantly in the DOX + SOP (10 mg/kg) group and the DOX + SOP (30 mg/kg) group in contrast to the DOX group (Fig. 1E–G). The results suggested that SOP could alleviated DOX-induced heart injury in mice.

### SOP inhibited DOX-induced oxidative stress in mice

**Figure 2:**
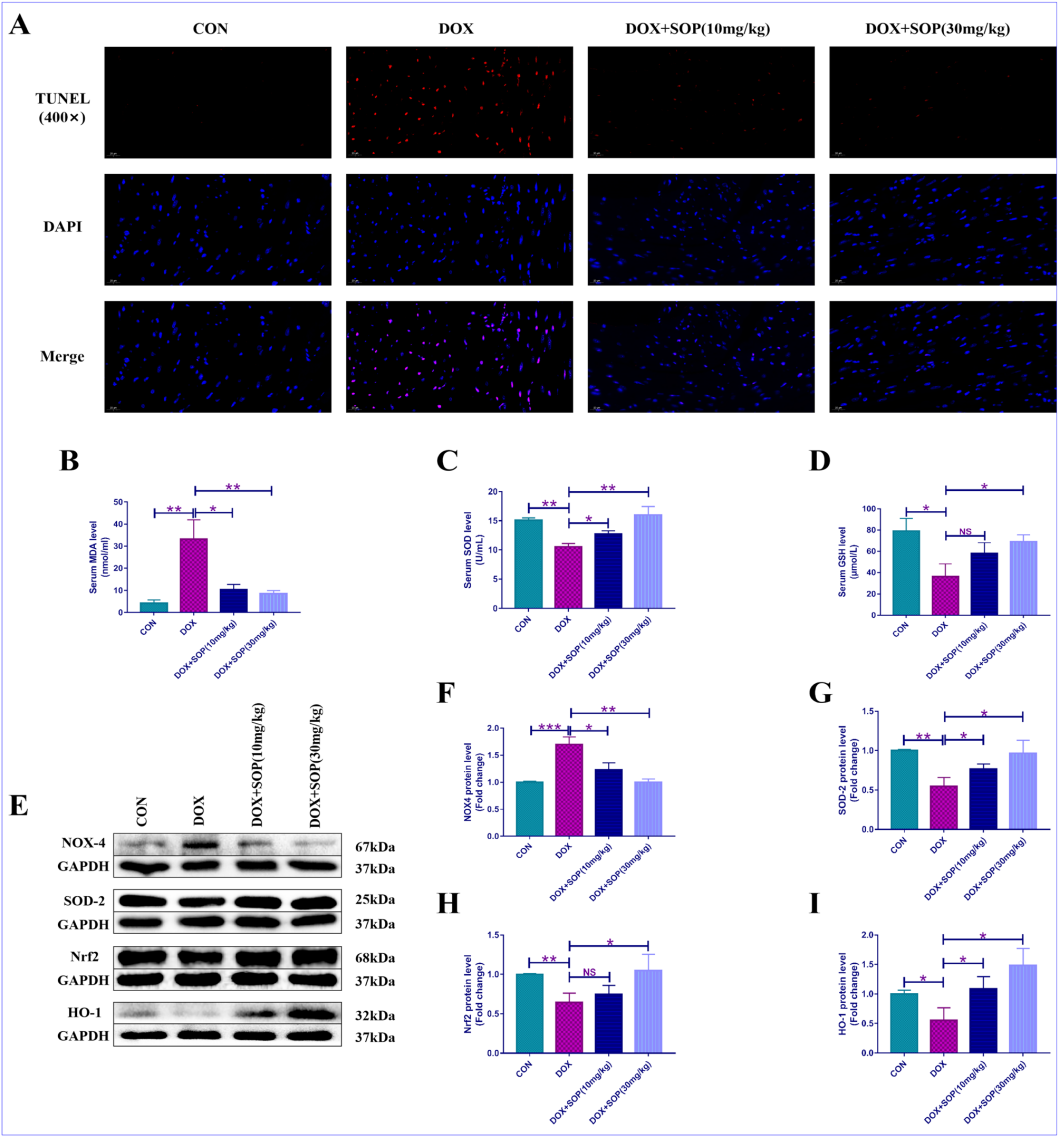
(**A**) DHE staining assay was conducted for evaluating oxidative stress level in heart tissues (n = 6). (**B**–**D**) Certain kits were applied to detect the serum levels of MDA, SOD and GSH (n = 6). (**E**–**G**) The protein expression levels of pro-oxidant and anti-oxidant proteins, NOX-4 and SOD-2, were detected by western blotting assay (n = 6). (**E**,**H**,**I**) The protein expression levels of Nrf2 and HO-1 were also detected by western blotting assay (n = 6). NS means no statistically significant; *p < 0.05, **p < 0.01 and ***p < 0.001. Data represent the mean ± SD of at least 3 times of separate experiments.

Oxidative stress degree in mice was assessed through DHE staining assay, oxidative stress biomarkers detection assay and western blotting assay. The degree of total superoxide evaluated by DHE staining assay was higher in the DOX group than the CON group, while it was inhibited in the DOX + SOP (10 mg/kg) group and the DOX + SOP (30 mg/kg) group in contrast to the DOX group (Fig. 2A). Compared with the CON group, the DOX group exerted a higher level of MDA and lower levels of SOD and GSH, while them were suppressed in the DOX + SOP (10 mg/kg) group and the DOX + SOP (30 mg/kg) group in contrast to the DOX group (Fig. 2B–D). Western blotting assay was applied for measuring pro-oxidative and anti-oxidative proteins, included NOX-4 and SOD-2. The results showed that DOX increased the level of NOX-4 while decreased the level of SOD-2 in the DOX group compared to the CON group, but SOP significantly reversed these changes in the DOX + SOP (10 mg/kg) group and the DOX + SOP (30 mg/kg) group in contrast to the DOX group (Fig. 2E–G). In addition, the present study further explored the effect of SOP on the activation of the nuclear factor erythroid 2-related factor-2 (Nrf2)/heme oxygenase-1 (HO-1) signaling pathway. The results revealed that DOX suppressed the Nrf2/HO-1 signaling pathway while SOP activated the Nrf2/HO-1 signaling pathway (Fig. 2E,H,I). The results indicated that SOP could suppress DOX induced oxidative stress through activating the Nrf2/HO-1 signaling pathway in mice.

### SOP hindered DOX-induced apoptosis in mice

**Figure 3:**
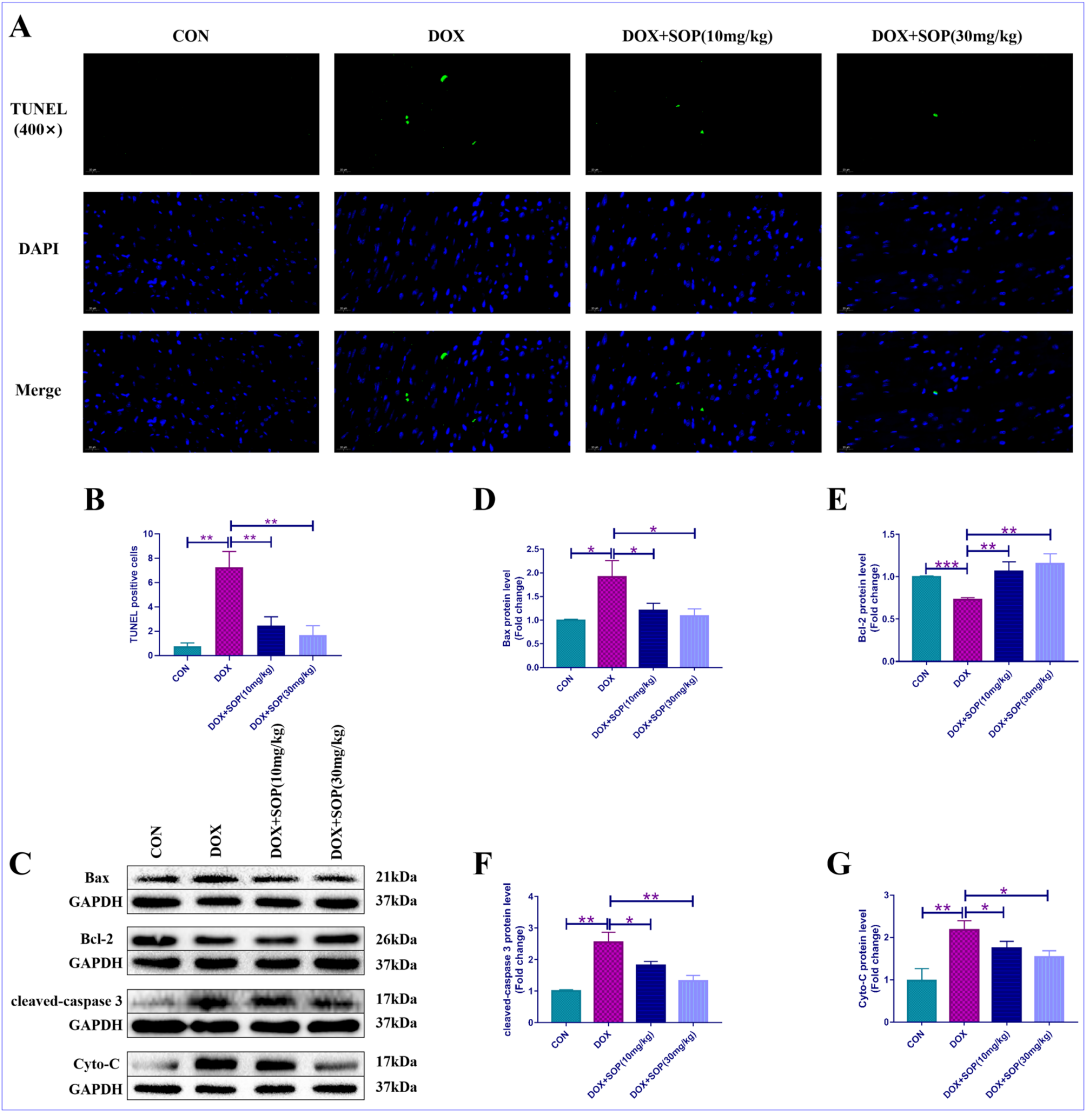
(**A**) and (**B**) TUNEL staining assay was conducted for evaluating apoptotic degree in heart tissues (n = 6). (**C**,**D**,**F**,**G**) The protein expression level of pro-apoptosis proteins, Bax, cleaved-caspase 3 and Cyto-C, were detected by western blotting assay (n = 6). (**C**) and (**E**), the protein expression levels of anti-apoptosis protein, like Bcl-2, was explored by western blotting assay (n = 6). *p < 0.05, **p < 0.01 and ***p < 0.001. Data represent the mean ± SD of at least 3 times of separate experiments.

The present study used TUNEL staining assay and western blotting assay to assess the degree of apoptosis in heart tissues. The TUNEL staining results revealed that the degree of apoptosis in the DOX group was higher than the CON group, but it was suppressed apparently in the DOX + SOP (10 mg/kg) group and the DOX + SOP (30 mg/kg) group in contrast to the DOX group (Fig. 3A and B). Besides, the present study also measured the expression levels of apoptosis-related proteins (Bax, Bcl-2, cleaved-caspase 3 and cytochrome-c (Cyto-C)). Compared with the CON group, the DOX group exerted a higher expression levels of Bax, cleaved-caspase 3 and Cyto-C and lower expression levels of Bcl-2, while them were reversed in the DOX + SOP (10 mg/kg) group and the DOX + SOP (30 mg/kg) group in contrast to the DOX group (Fig. 3C–G). The results revealed that SOP could inhibit DOX induced apoptosis in mice.

### SOP suppressed DOX-induced oxidative stress in H9C2 cells

**Figure 4:**
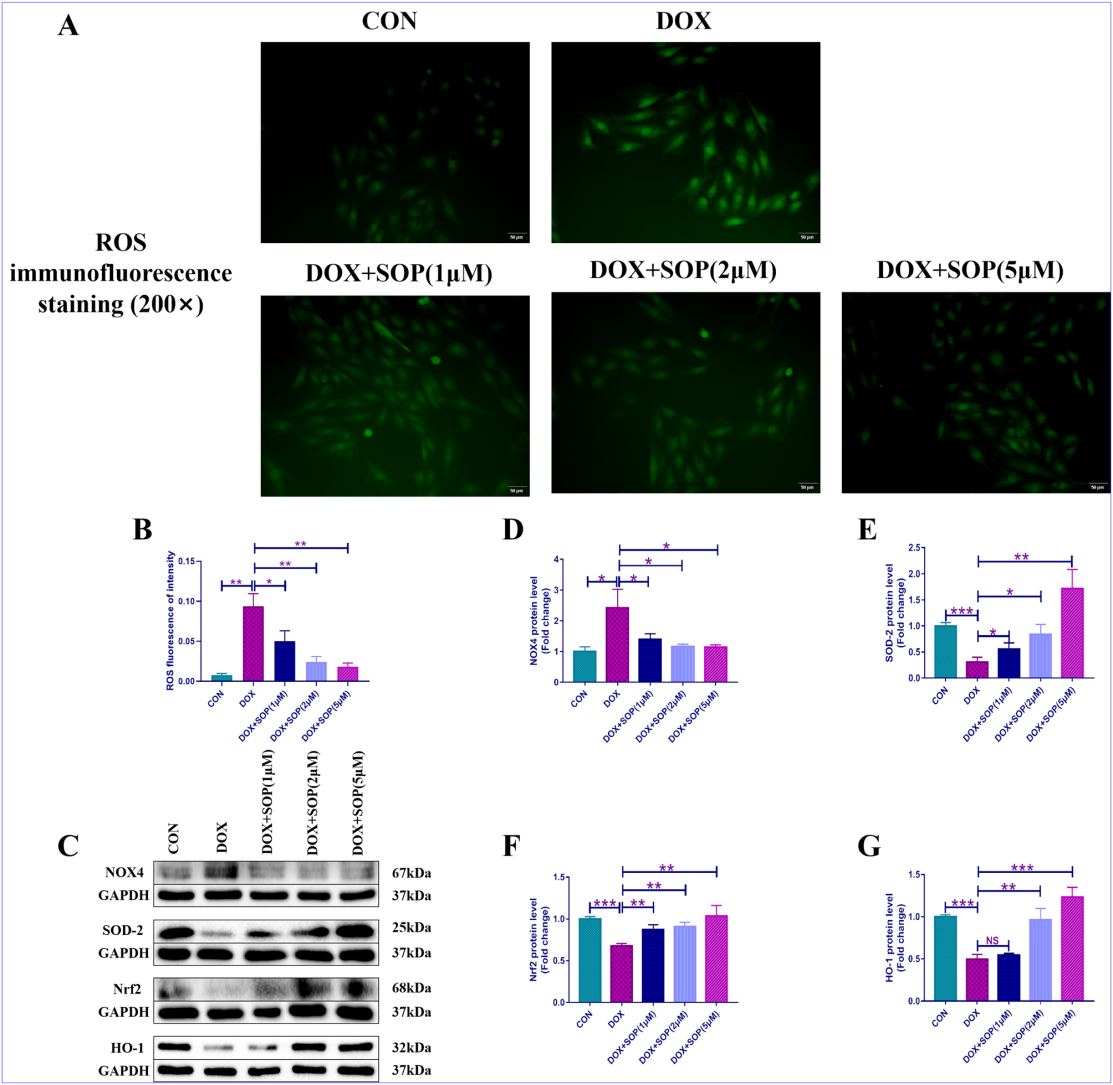
(**A**) and (**B**), ROS kit was used to evaluate oxidative stress level in H9C2 cells (n = 6). (**C**) and (**D**), the protein expression levels of pro-oxidant protein, NOX-4, was detected by western blotting assay (n = 6). (**C**) and (**E**), the protein expression levels of anti-oxidant protein, SOD-2, was detected by western blotting assay (n = 6). (**C**,**F**,**G**), the protein expression levels of Nrf2 and HO-1 were also detected by western blotting assay (n = 6). NS means no statistically significant; *p < 0.05, **p < 0.01 and ***p < 0.001. Data represent the mean ± SD of at least 3 times of separate experiments.

Compared with the CON group, the fluorescence intensity measured through ROS kit apparently upregulated in the DOX group, but SOP significantly suppressed this change in the DOX + SOP (1 μM, 2 μM, 5 μM) groups in contrast to the DOX group (Fig. 4A and B). The present study further detected the pro-oxidative and anti-oxidative proteins, included NOX-4 and SOD-2. The results revealed that DOX increased the expression level of NOX-4 while decreased the expression level of SOD-2 in the DOX group compared to the CON group, but SOP markedly reversed these changes in the DOX + SOP (1 μM, 2 μM, 5 μM) groups in contrast to the DOX group (Fig. 4C–E). Besides, the present study further investigated the effect of SOP on the activation of the Nrf2/HO-1 signaling pathway. The results showed that DOX inhibited the Nrf2/HO-1 signaling pathway while SOP activated the Nrf2/HO-1 signaling pathway (Fig. 4C,F,G). The results suggested that SOP could inhibit DOX induced oxidative stress through activating the Nrf2/HO-1 signaling pathway in H9C2 cells.

### SOP reduced DOX-induced apoptosis in H9C2 cells

**Figure 5:**
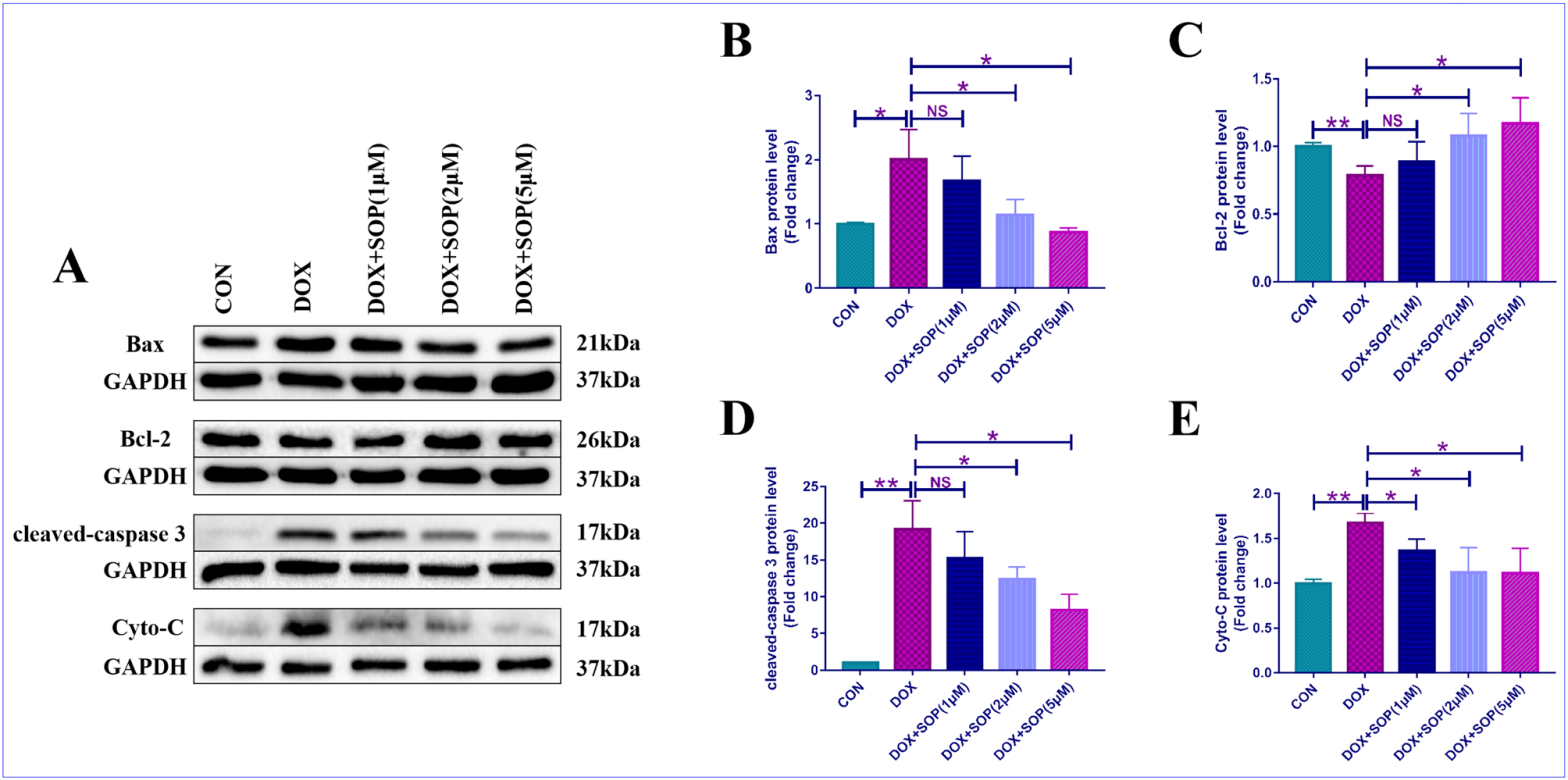
(**A**,**B**,**D**,**E**) the protein expression levels of pro-apoptosis proteins, like Bax, cleaved-caspase 3 and Cyto-C, were explored by western blotting assay in H9C2 cells (n = 6). (**A**) and (**C**), the protein expression level of anti-apoptosis protein, like Bcl-2, was also detected by western blotting assay (n = 6). NS means no statistically significant; *p < 0.05 and **p < 0.01. Data represent the mean ± SD of at least 3 times of separate experiments.

Western blotting assay was applied for assessing the degree of apoptosis in H9C2 cells. The present study measured the expression levels of apoptosis-related proteins (Bax, Bcl-2, cleaved-caspase 3 and cytochrome-c (Cyto-C)). Compared with the CON group, the DOX group exerted a higher expression levels of Bax, cleaved-caspase 3 and Cyto-C and lower expression levels of Bcl-2, while them were reversed in the DOX + SOP (1 μM, 2 μM, 5 μM) groups in contrast to the DOX group (Fig. 5A–E). All the results indicated that SOP could reduce DOX induced apoptosis in H9C2 cells.

## Discussion

**Figure 6:**
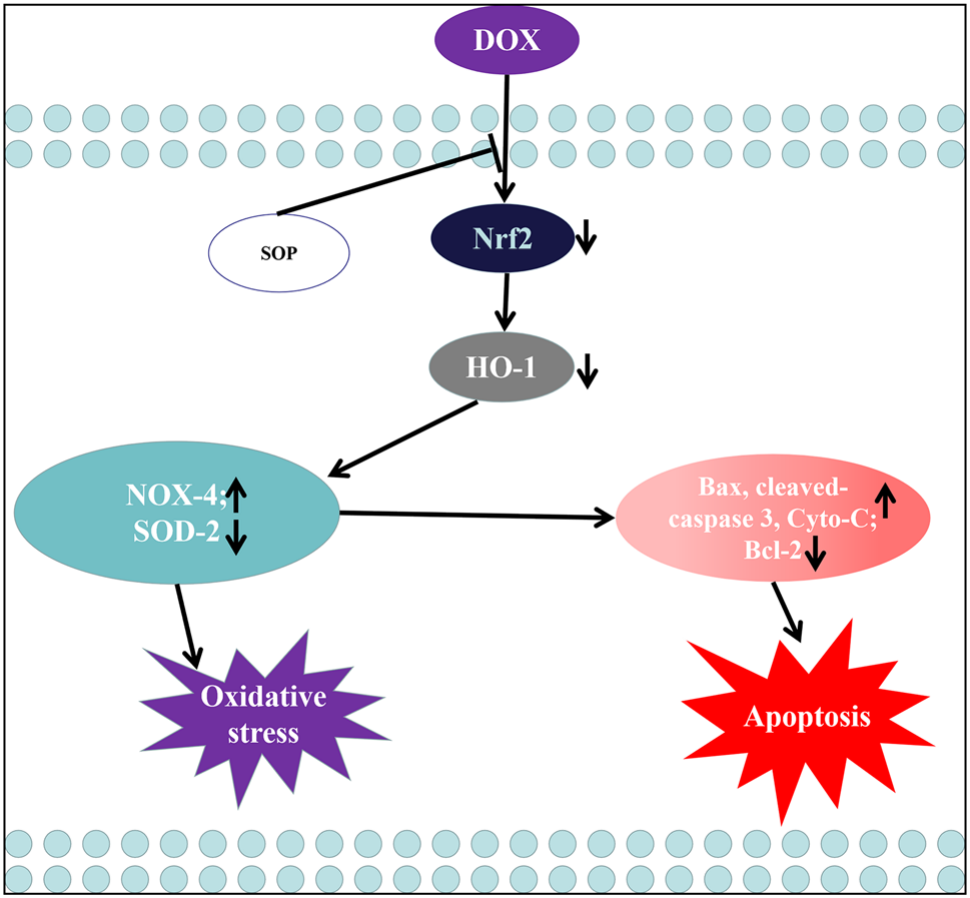
Graphical depiction of sophocarpine treatment effect on DOX induced heart injury.

DOX is a highly effective anthracycline antibiotic against tumors, currently the DOX based chemotherapy remains the cornerstone of cancer treatment^14^. However, it’s dose-dependent heart injury greatly limit its application in clinical practice^15^. While, there is no good treatment that can alleviate DOX-induced heart injury^16^. Therefore, exploring and developing safe and effective treatments to alleviate DOX-induced heart injury is of great significance. SOP is a type of a tetracyclic quinolizidine alkaloid extracted from the traditional chinese medicine sophora flavescens^17^, which exerts various biological effects in vivo like anti-nociceptive, anti-inflammatory, anti-oxidative stress, neuroprotective, immune regulatory functions, and anti-tumor^12^. Our previous research revealed that SOP can alleviate heart injury induced by lipopolysaccharide, and further found that SOP exerts its cardioprotective effect by inhibiting inflammation, apoptosis, oxidative stress and autophagy^13^. So we speculate that SOP can also alleviate DOX-induced heart injury, and found that SOP could alleviate DOX-induced heart injury by inhibiting oxidative stress and apoptosis (Fig. 6).

Oxidative stress refers to the imbalance between oxidation reaction and reduction reaction in the body, which result in the production of a large number of intermediate oxidation products and further lead to multiple organs damage^18^. Oxidative stress was reported to be one of major pathological changes in DOX-induced heart injury^19^. When the degree of oxidative stress upregulates apparently, the level of malondialdehyde (MDA) increases while the levels of superoxide dismutase (SOD) and glutathione (GSH) decrease^20^. NOX-4 is an oxidase, which can exacerbate levels of oxidative stress^21^. While SOD-2 is an antioxidant enzyme that can reduce the level of oxidative stress^22^. In addition, oxidative stress is usually regulated by the Nrf2/HO-1 signaling pathway^23^. The present study found that SOP could alleviate DOX induced oxidative stress in mice and H9C2 cells. Firstly, The results of DHE and ROS staining assay showed that SOP reversed the increases of total superoxide level induced by DOX both in mice and H9C2 cells. Then, DOX significantly contributed to a higher level of MDA and lower levels of SOD and GSH, but these effects were suppressed by SOP in mice. Next, the present study discovered that DOX significantly increased the pro-oxidative protein expression level of NOX-4 while decreased the anti-oxidative protein expression level of SOD-2, while SOP reversed these effects both in mice and H9C2 cells. In addition, we further measured the effects of SOP on the activation of the Nrf2/HO-1 signaling pathway, and results revealed that SOP inhibited the decreases of Nrf2 and HO-1 levels induced by DOX both in mice and H9C2 cells. Thus, the present study concluded that SOP could alleviate DOX induced oxidative stress by activating the Nrf2/HO-1 signaling pathway in mice and H9C2 cells.

Apoptosis refers to the autonomous and orderly death of cells controlled by genes to maintain the stability of the internal environment^24^. But when the excessive apoptosis could contribute to damage to the corresponding tissues^25^. Apoptosis was also reported to be one of major pathological changes in DOX-induced heart injury^26^. The results of TUNEL staining assay suggested that SOP reduced the degree of apoptosis induced by DOX in mice. The present study further found that the levels of pro-apoptosis proteins (Bax, cleaved-caspase 3 and Cyto-C) upregulated while the level of anti-apoptosis protein (Bcl-2) downregulated when mice and H9C2 cells were challenged by DOX, but these effects were reversed by SOP in mice and H9C2 cells. The present study discovered that SOP could attenuate DOX induced apoptosis in mice and H9C2 cells.

However, this investigation could not explain the exact molecular mechanism; therefore, more research and experiments are required for an in-depth investigation to efficiently elaborate the pathogenesis of DOX induced heart injury, like using inhibitors of the Nrf2/HO-1 signaling pathway to verify the accuracy of the molecular mechanism (Supplementary Information).

## Conclusion

The present study showed that SOP could alleviate DOX-induced heart injury by inhibiting oxidative stress and apoptosis,. The molecular mechanism was the activation of the Nrf2/HO-1 signaling pathway, indicating that SOP acts as a novel therapeutic method for DOX-induced heart injury.

### Supplementary Information

Supplementary Information.

## Data Availability

The datasets generated during the present study are available from the corresponding author on reasonable request.
