# Supplementary material for: Sophocarpine alleviates doxorubicin-induced heart injury by suppressing oxidative stress and apoptosis
Source: Sci Rep. 2024 Jan 3;14:428. doi: 10.1038/s41598-023-51083-3 (PMC10764776; doi:10.1038/s41598-023-51083-3)

Gel images： It should be noted that the blots in the present study were cut prior to hybridisation with antibodies during the western blotting. And we added it to the method section of our manuscrpt.

Mice:

NOX-4: 67 kDa GAPDH: 37 kDa

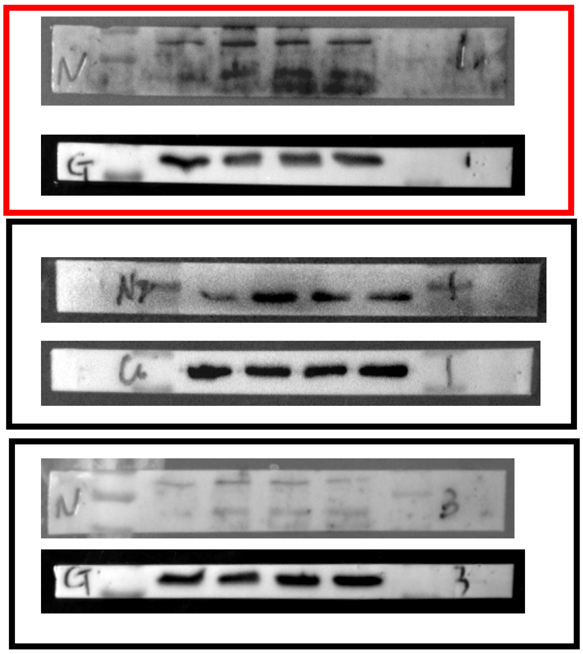

SOD-2: 25 kDa GAPDH: 37 kDa

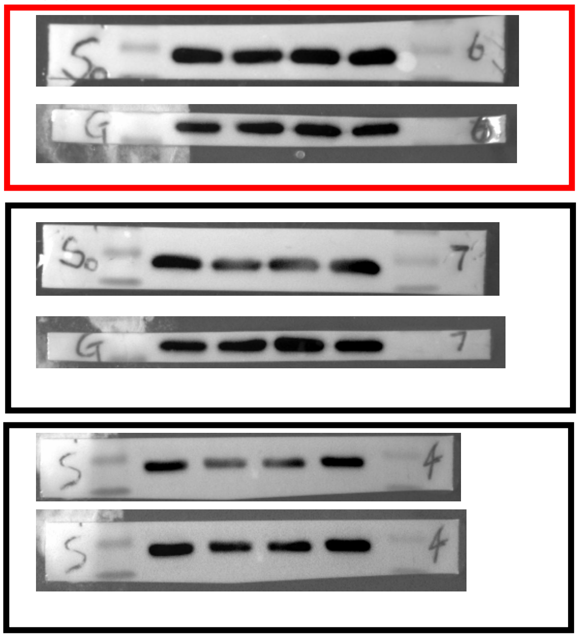

Nrf2：68 kDa GAPDH: 37 kDa

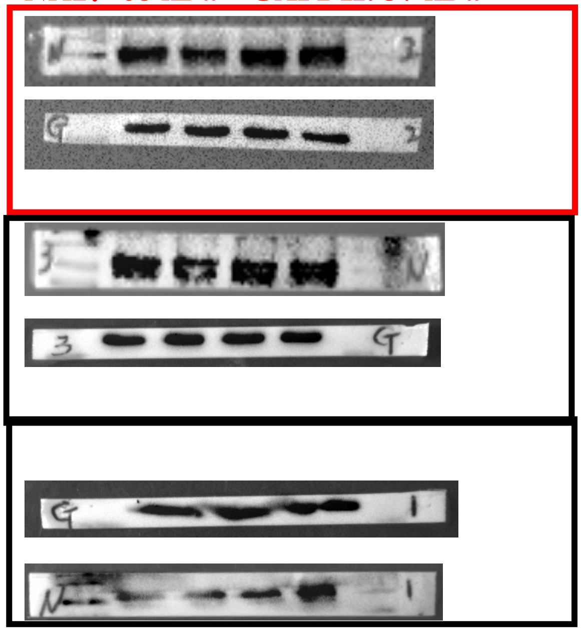

HO-1: 32 kDa GAPDH: 37 kDa

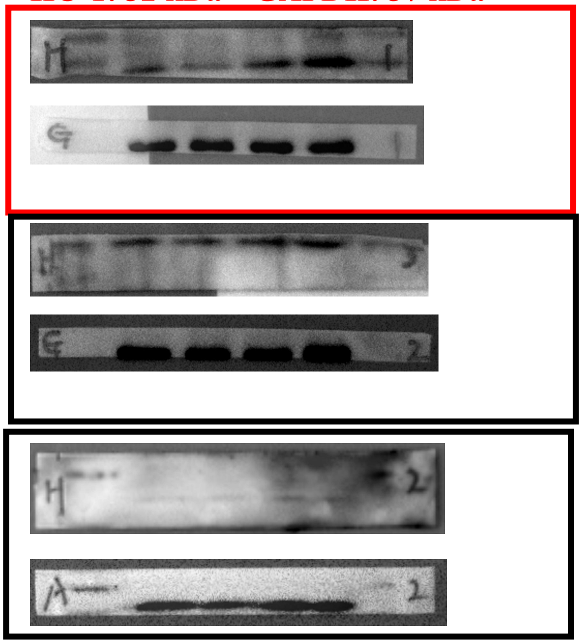

Bax：21 kDa   GAPDH: 37 kDa

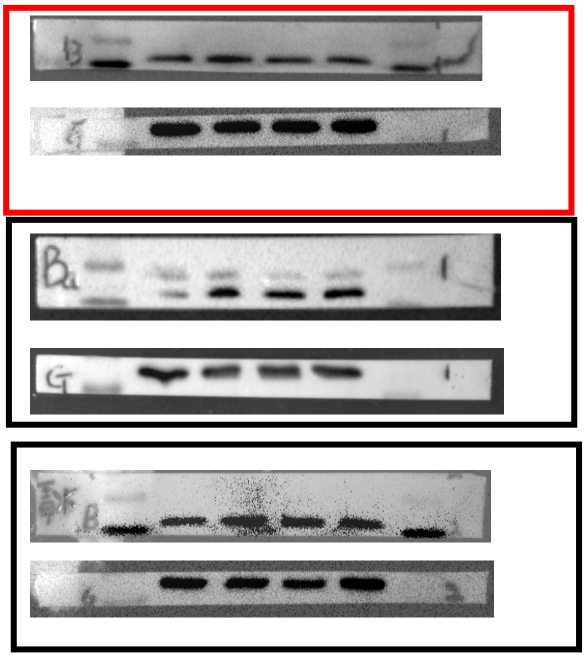

BCL-2: 26 kDa   GAPDH: 37 kDa

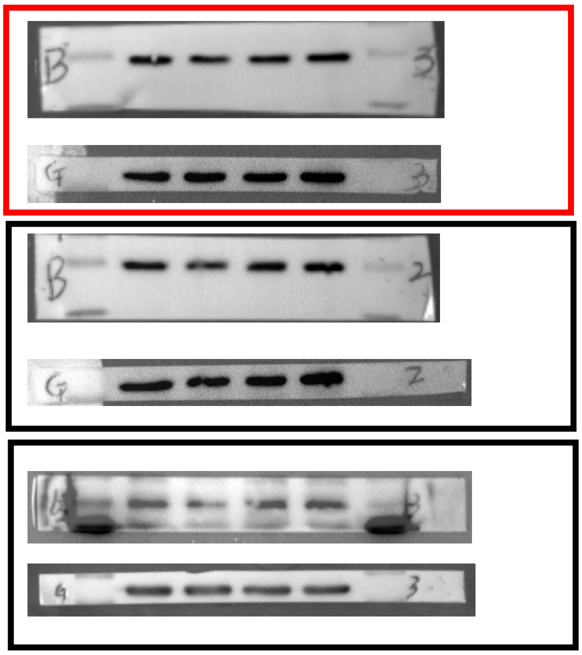

cleaved-caspase 3：17 kDa GAPDH: 37 kDa

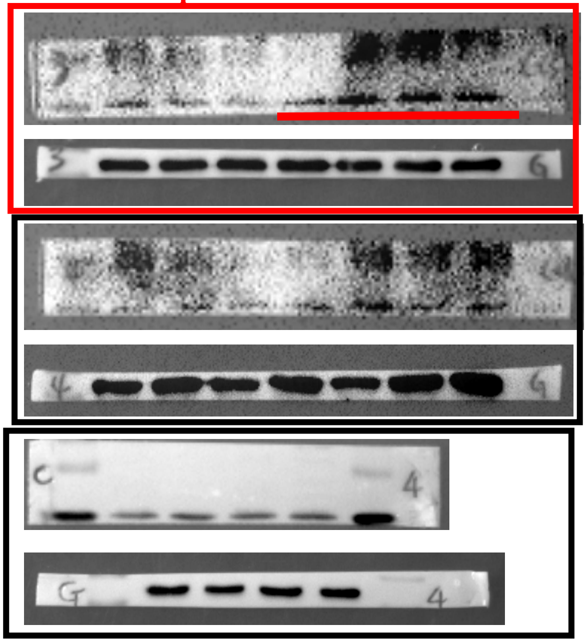

Cyto-C: 17 kDa GAPDH: 37 kDa

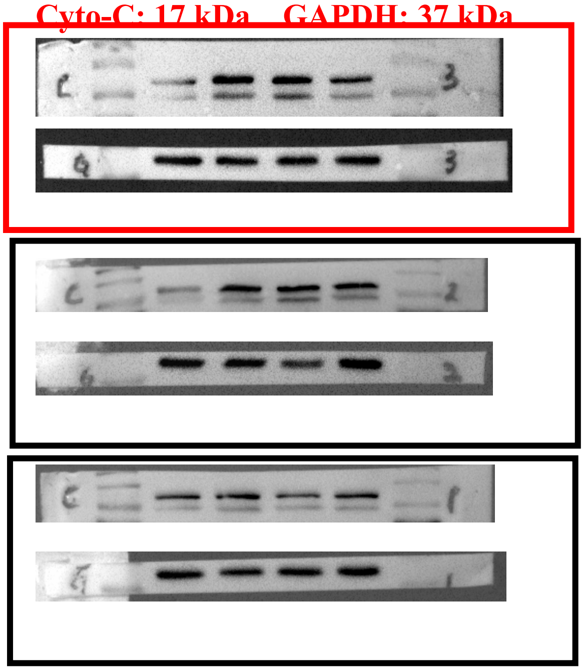

H9C2 cells:

NOX-4: 67 kDa GAPDH: 37 kDa

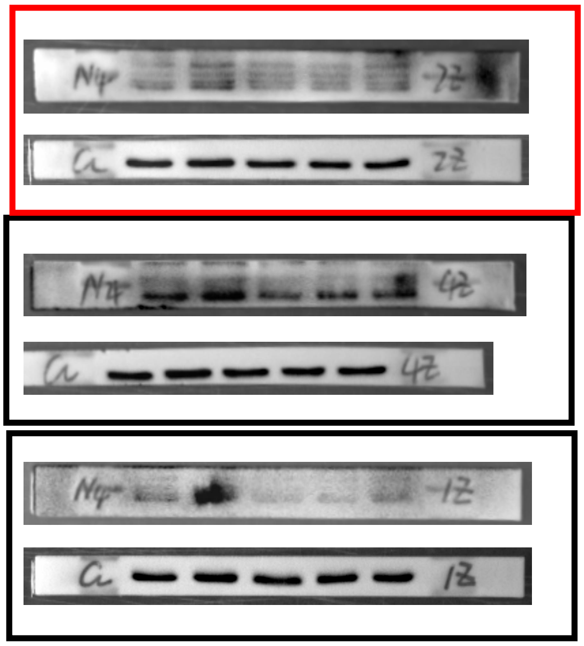

SOD-2: 25 kDa GAPDH: 37 kDa

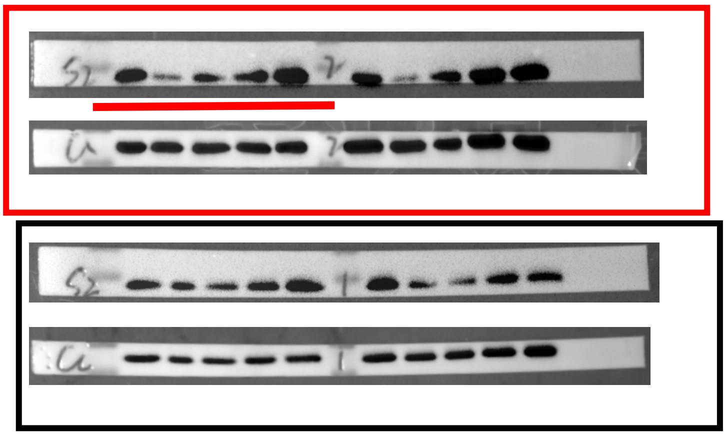

Nrf2：68 kDa GAPDH: 37 kDa

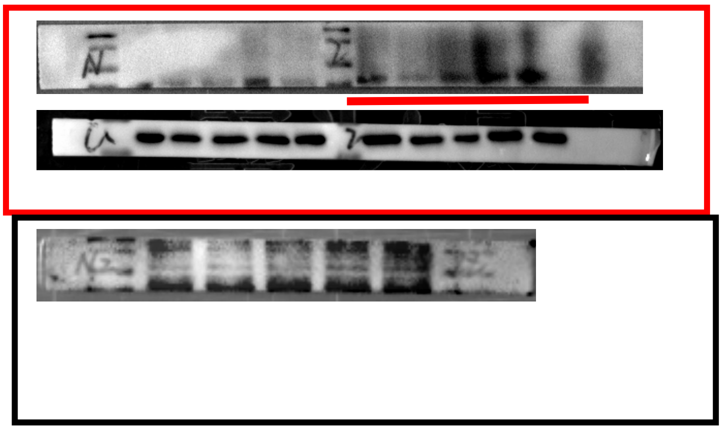

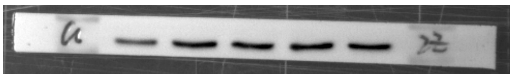

HO-1: 32 kDa   GAPDH: 37 kDa

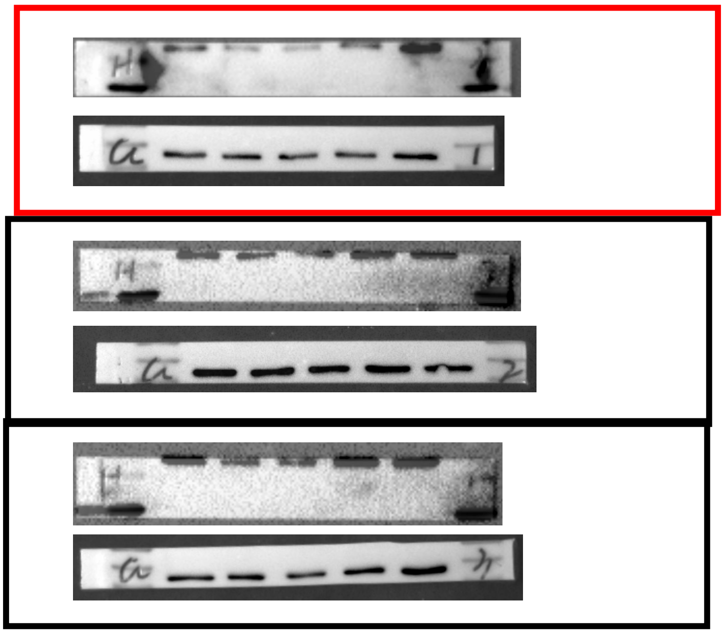

Bax：21 kDa   GAPDH: 37 kDa

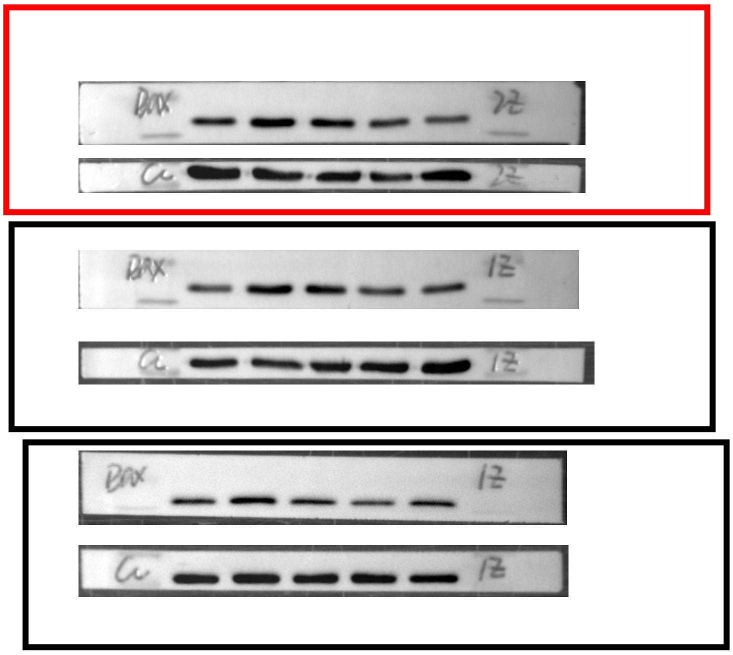

BCL-2: 26 kDa   GAPDH: 37 kDa

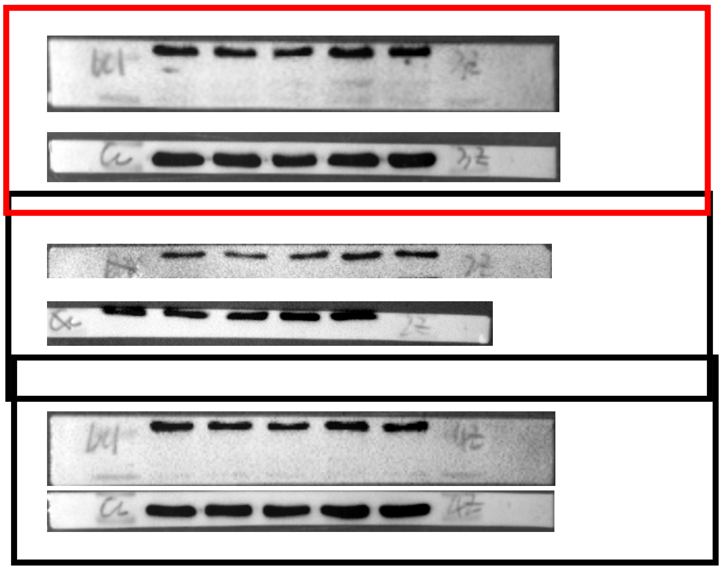

cleaved-caspase 3：17 kDa   GAPDH: 37 kDa

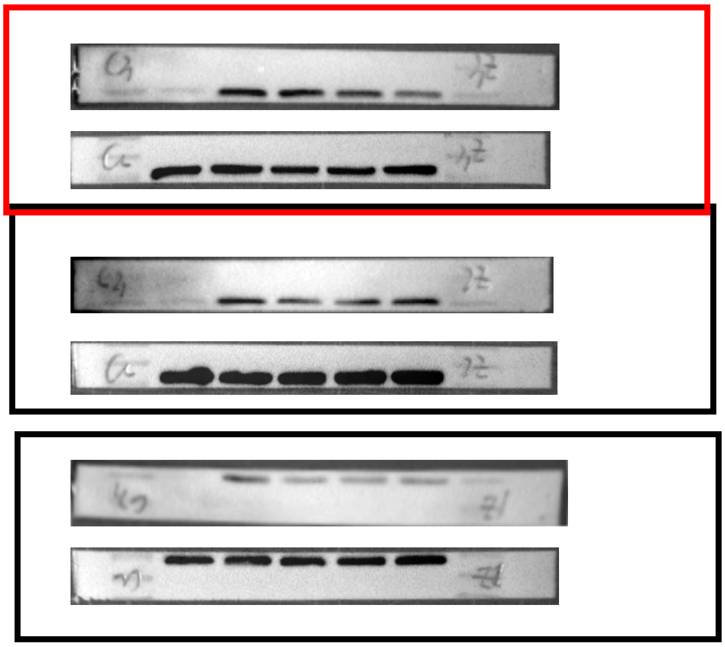

Cyto-C: 17 kDa GAPDH: 37 kDa

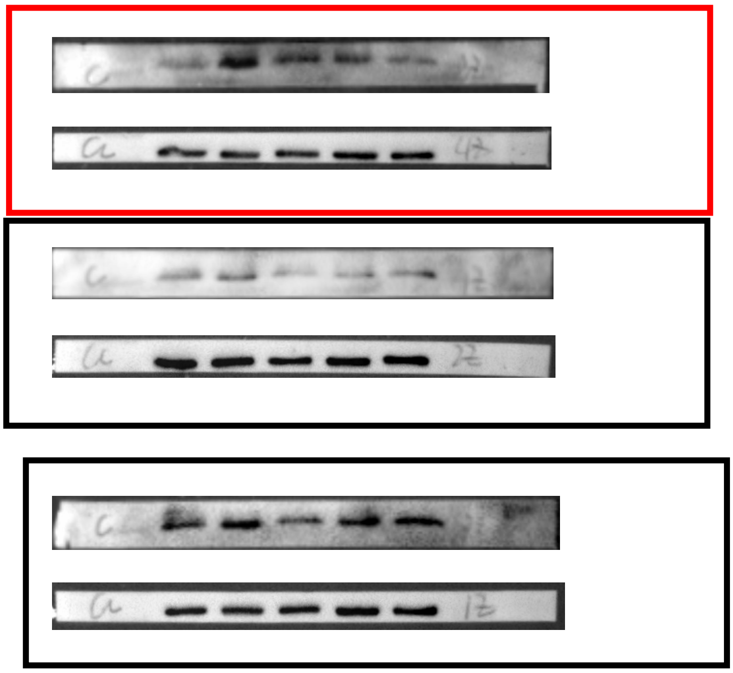

Supplement: Supplementary file 1 — Supplementary Information. [file 41598_2023_51083_MOESM1_ESM.pdf]
